# Clinical and Digital Workflow for an Implant-Retained Overdenture in a Regenerated Maxilla: A Case Report

**DOI:** 10.7759/cureus.117600

**Published:** 2026-10-07

**Authors:** María de Lourdes Bravo Guerrero, Pedro Alberto López Reynoso, Enrique Ríos Szalay, Daniela Carmona Ruiz, Cesar Esquivel Chirino

**Affiliations:** 1 Advanced Restorative Dentistry, División de Estudios de Posgrado e Investigación, Facultad de Odontología, Universidad Nacional Autónoma de México, Mexico City, MEX; 2 Periodontics and Implantology Specialty, División de Estudios de Posgrado e Investigación, Facultad de Odontología, Universidad Nacional Autónoma de México, Mexico City, MEX; 3 Orthodontics, Facultad de Odontología, Universidad Nacional Autónoma de México, Mexico City, MEX; 4 Basic Medical Sciences, Facultad de Odontología, Universidad Nacional Autónoma de México, Mexico City, MEX

**Keywords:** alveolar ridge preservation, bone graft, digital planning, guided bone regeneration, overdenture, tent-pole technique

## Abstract

The rehabilitation of edentulous jaws with severe bone resorption represents a clinical challenge. Among the available therapeutic options, guided bone regeneration (GBR), complemented by precise digital planning, can help create more favorable conditions for implant placement and subsequent prosthetic rehabilitation. Following tooth extraction, substantial alveolar bone loss can occur during the first six months, progressively altering the intermaxillary relationships.

We report the case of a 59-year-old female patient with a Seibert Class III defect in the anterior maxilla, a pseudo-skeletal Class III intermaxillary relationship, and stage III, grade C periodontitis. Alveolar ridge preservation was performed in the posterior maxillary sites, and GBR using the tent-pole technique was carried out in the anterior sector with a combination of xenograft, allograft, and autograft covered by a porcine collagen membrane. Implant planning followed a dual-scan cone-beam computed tomography (CBCT) protocol. Baseline and nine-month CBCT superimposition revealed regional dimensional changes: in the ridge-preservation sites, a mean crestal resorption of 1.55 mm at 1 mm from the crest coexisted with an apical gain of 0.86 mm at 5 mm; in the tent-pole area, a horizontal increase of up to 3.36 mm was observed, with concomitant vertical loss. In the mandible, a xenograft alone was associated with favorable osseointegration of the subsequently placed implant. Prosthetically guided planning articulated the surgical and prosthetic phases and allowed completion of rehabilitation with an implant-retained, bar-supported overdenture. Long-term functional and esthetic evaluations remain ongoing. Within the limitations of a single case, the combined regenerative and digital workflow was associated with clinically favorable outcomes.

## Introduction

After dental extraction, physiological remodeling of the alveolar ridge occurs, with a horizontal reduction that can reach between 29% and 63% of the original width and an approximate vertical loss of 1-2 mm during the first months of healing [1]. Resorption is more pronounced on the buccal wall, which has a thinner cortical plate that resorbs more than the palatal and lingual walls [2]. Alveolar ridge preservation aims to slow the contraction and collapse of the ridge after extraction.

These resorption patterns progressively alter the intermaxillary relationships. In edentulous patients with severe maxillary atrophy, a clinical appearance resembling a skeletal Class III relationship (pseudo-Class III) may develop, characterized by a relatively forward position of the mandible with respect to the maxilla, which can complicate prosthetic planning and rehabilitation [3]. In terms of anatomical severity, alveolar ridge deficiencies are commonly classified according to Seibert as Class I (horizontal loss), Class II (vertical loss), or Class III (combined horizontal and vertical deficiency), the latter representing the most demanding scenario for implant-supported rehabilitation.

When resorption prevents the placement of the implant in its ideal three-dimensional position, bone regeneration becomes a suitable alternative. The objective is to generate a bone volume that houses the implant in a three-dimensionally stable position, supports the surrounding soft tissue, and offers a favorable long-term prognosis. Available strategies include block grafts, guided bone regeneration (GBR), ridge expansion, and distraction osteogenesis [4]. GBR is a well-established regenerative approach that relies on the use of a barrier membrane to protect the graft from soft-tissue invasion and to allow undisturbed bone formation within the defect.

The tent-pole technique involves placing screws that form a tent-like structure, which maintains the space and prevents the collapse of the grafted area [5].

With respect to the surgical guide, for edentulous jaws, mucosa- or bone-supported guides are indicated, and their fabrication is primarily based on the dual-scan protocol. In brief, the dual-scan protocol consists of two cone-beam computed tomography (CBCT) acquisitions: one of the patient wearing a radiographic guide with radiopaque markers and one of the guide alone, which are subsequently superimposed in the planning software to enable prosthetically driven implant positioning [6,7].

In severely resorbed maxillae, both fixed full-arch and implant-retained removable prostheses may be considered. Implant-retained overdentures remain a valuable option in this scenario because the acrylic flange can compensate for the volumetric loss of soft tissue, restore lip support and the vertical dimension of occlusion (VDO), and simplify hygiene access around the implants; these considerations are particularly relevant in patients with a history of periodontal susceptibility or previous implant failure. The patient-specific rationale for this decision is discussed in the Case Presentation.

The present case report describes the combined regenerative and digital workflow used to rehabilitate a severely resorbed maxilla with a Seibert Class III defect and a pseudo-skeletal Class III intermaxillary relationship in a patient with previous implant failure and a periodontally susceptible dentition. Its clinical significance lies in illustrating how alveolar ridge preservation, tent-pole GBR, and prosthetically guided digital planning can be integrated to enable an implant-retained overdenture in a scenario that would otherwise pose considerable prosthetic difficulty. The case is offered as an educational reference for clinicians facing similar situations.

## Case presentation

A 59-year-old female patient was referred to the Advanced Restorative Dentistry Clinic of the School of Dentistry, Universidad Nacional Autónoma de México (UNAM), for functional and esthetic rehabilitation of a partially edentulous, severely resorbed maxilla. Her medical history was non-contributory, with no relevant systemic conditions, allergies, or long-term medications reported. Her dental history was significant for previous orthognathic surgery and two prior GBR procedures in the anterior maxillary sector, together with multiple missing teeth in the anterior maxilla, a previously placed implant at site #46 that had shown clinical and radiographic signs of failure, and a chronically compromised mandibular right second molar (#47). She reported difficulty with mastication, phonetics, and esthetics due to the loss of anterior teeth and the collapse of the alveolar ridge. Written informed consent was obtained, authorizing the diagnostic procedures, the surgical and prosthetic treatment, and the anonymized use of her clinical records and images for academic and scientific purposes.

Extraoral examination showed a decreased lower facial third and reduced upper lip support, consistent with severe anterior maxillary bone resorption, and the patient presented with an altered occlusal scheme. Clinical assessment of the VDO, performed with phonetic, esthetic, and interocclusal rest position tests, revealed loss of VDO secondary to tooth loss, ridge collapse, and attrition of the residual dentition; this loss required re-establishment of the VDO through the new rehabilitation. Intraoral examination revealed a Seibert Class III (combined horizontal and vertical) defect in the anterior maxilla, involving sites #11, #21, #22, and #23, together with additional edentulous areas in the posterior maxilla, several teeth without adequate prosthetic value, and a periodontal status consistent with stage III, grade C periodontitis. CBCT confirmed the severity of the horizontal and vertical ridge deficiency in the anterior maxilla and a bone volume that was insufficient for prosthetically driven implant placement without prior augmentation (Figure 1).

**Figure 1:**
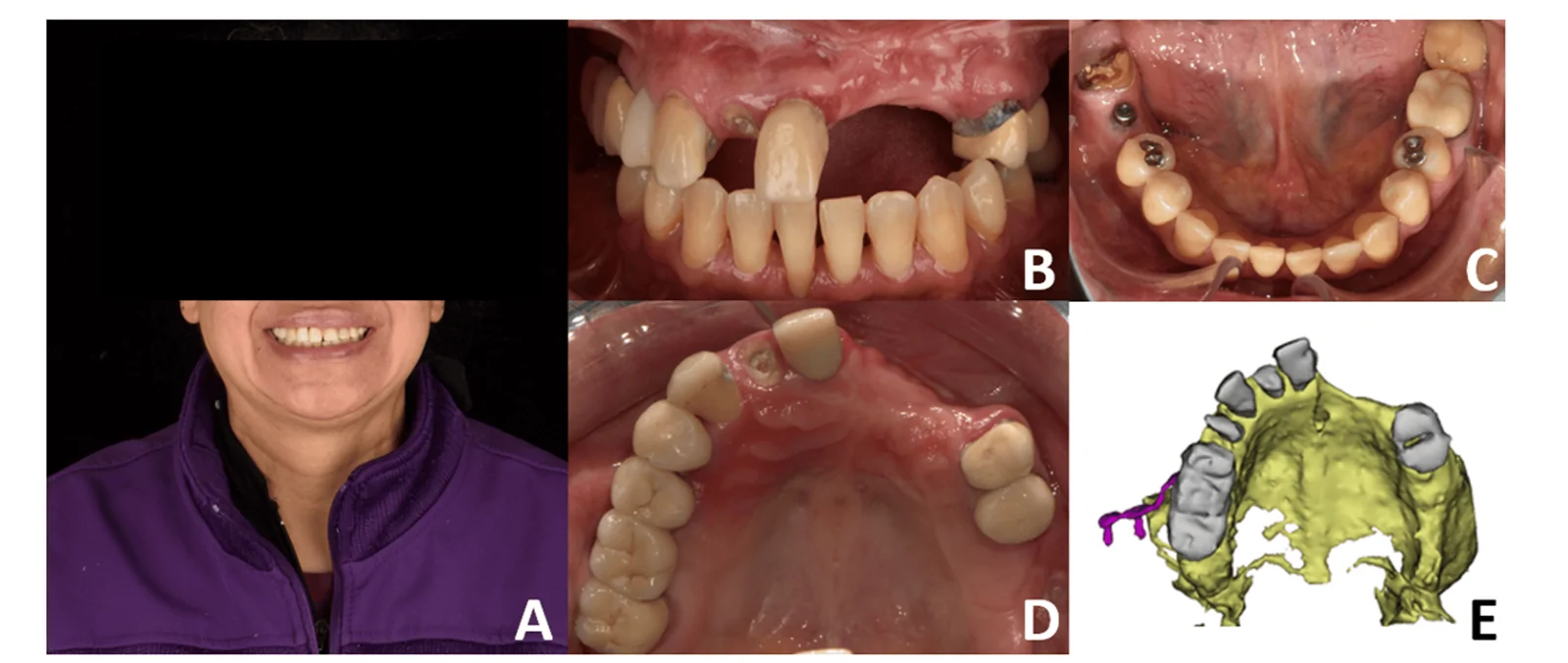
Initial clinical and tomographic images. (A) Extraoral photograph; (B-D) intraoral photographs illustrating the anterior maxillary edentulous area, the residual dentition, and the Angle Class III intermaxillary relationship; (E) tomographic segmentation of the Seibert Type III defect in the anterior maxilla.

The Class III intermaxillary relationship had been prosthetically compensated by the existing restorations; however, these restorations were in poor condition, no longer offered adequate prosthetic value, and were therefore considered non-conservable. The failing implant at site #46 exhibited mobility and peri-implant bone loss, and tooth #47 presented with a poor restorative and periodontal prognosis; a complete preoperative periapical radiographic series was obtained to document the status of the residual dentition, the failing implant at site #46, and the compromised tooth #47 (Figure 2).

**Figure 2:**
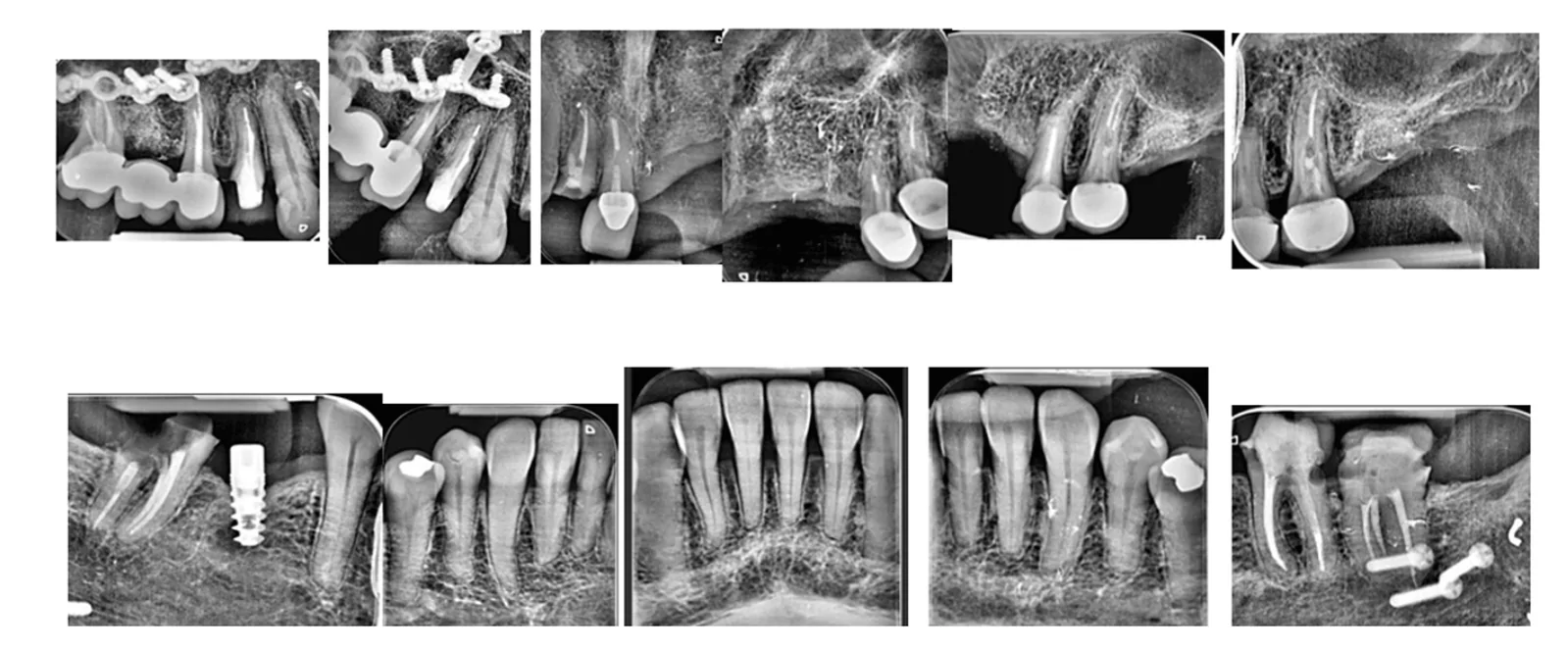
Preoperative periapical radiographic series documenting the status of the residual dentition, the failing implant at site #46, and the compromised tooth #47. Upper row: maxillary sextants, from the right posterior area to the left posterior area, showing the residual roots, the anterior maxillary edentulous span, and the marginal condition of the remaining maxillary teeth. Lower row: mandibular sextants, from the right posterior area to the left posterior area, showing the peri-implant bone loss around the failing implant at site #46, the periodontal and restorative status of tooth #47, and the condition of the remaining mandibular teeth.

The intermaxillary relationship was additionally documented by mounting diagnostic casts on a semi-adjustable articulator, which confirmed the Angle Class III occlusion (Figure 3).

**Figure 3:**
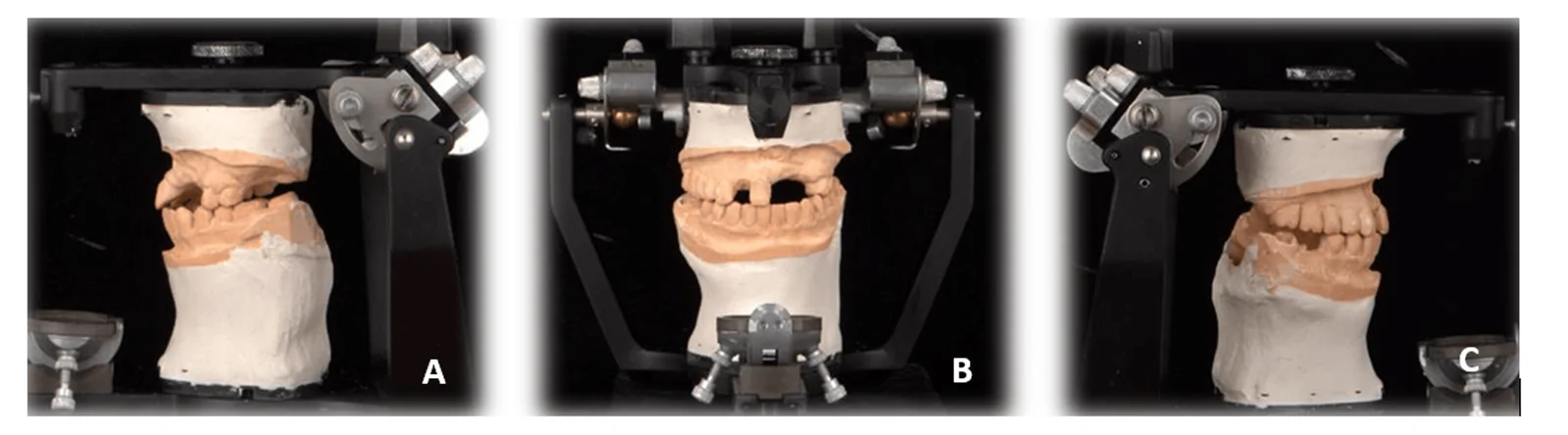
Diagnostic casts mounted on a semi-adjustable articulator, illustrating the preoperative intermaxillary relationship. (A) Right lateral view, (B) frontal view, (C) left lateral view. The mounted casts objectively confirm the Angle Class III intermaxillary relationship and the decreased vertical dimension of occlusion described clinically.

Based on these findings, a staged, prosthetically driven treatment plan was accepted by the patient, consisting of (i) alveolar ridge preservation of the posterior maxillary post-extraction sites and mandibular ridge preservation after explantation of implant #46 and extraction of tooth #47; (ii) tent-pole GBR of the Seibert Class III anterior maxillary defect; (iii) prosthetically guided implant planning based on a dual-scan CBCT protocol; and (iv) definitive rehabilitation with an implant-retained, bar-supported overdenture in the maxilla and screw-retained zirconia crowns in the mandible.

The decision to rehabilitate the maxilla with an implant-retained, bar-supported overdenture rather than a fixed full-arch prosthesis was based on four patient-specific factors: (i) the need for adequate lip and midfacial support, which was to be compensated by incorporating a buccal flange in the removable prosthesis; (ii) hygienic considerations, since, in the presence of a Class III intermaxillary relationship, a buccal flange placed in a fixed prosthetic rehabilitation could favor plaque accumulation and hinder adequate hygiene; (iii) biomechanical control, since the implant-retained overdenture allowed a design aimed at achieving a more controlled distribution of functional loads, taking into account the intermaxillary relationship and the position of the implants; and (iv) removability, which facilitates access for oral hygiene and long-term maintenance of the peri-implant tissues by both the patient and the clinical team. The available prosthetic space was sufficient for either a fixed or a removable rehabilitation and was therefore not the decisive factor in this choice.

Because the initial bone volume of the anterior maxilla did not allow prosthetically driven implant placement, implants could not be inserted in this first surgical phase. Ridge preservation was performed at the post-extraction sites with an allograft (OraGRAFT 70/30 Cortical, LifeNet Health, Virginia Beach, Virginia, USA) mixed with an autograft from the anterior nasal spine, and tent-pole GBR was carried out using two 2 × 10 mm screws placed at 45° in sites #11, #21, #22, and #23. As a biomaterial, a xenograft (InterOss 0.25-1 mm; SigmaGraft, Fullerton, California, USA) mixed with an allograft (OraGRAFT 70/30 Cortical) and an autograft from the same area was used. The graft was covered with a porcine collagen membrane (Intercollagen Guide 30 × 40 mm, SigmaGraft) fixed with tacks (AutoTac; BioHorizons, Birmingham, Alabama, USA). After the periosteum was released to achieve tension-free closure, the surgical site was sutured with 4-0 nylon using horizontal mattress and interrupted sutures (Figure 4).

**Figure 4:**
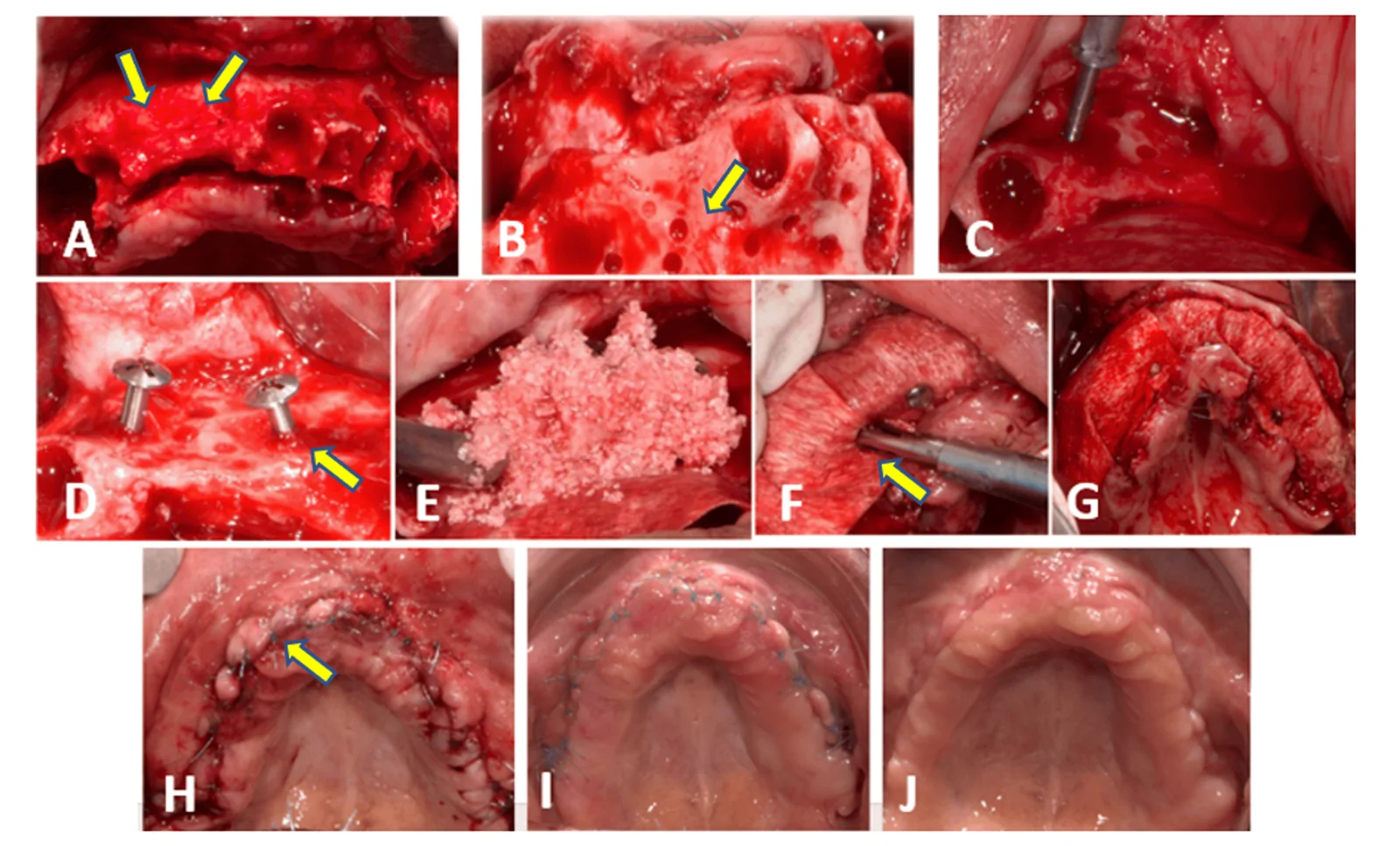
Tent-pole guided bone regeneration. (A) Full-thickness flap; the arrow indicates the Seibert Type III defect in the anterior region. (B) Cortical perforations; the arrow indicates the holes created for adequate irrigation of the bone graft. (C) Screw placement; the arrow indicates the placement of the screws at a 45-degree angulation. (D) Screws in the inclined arrangement; the arrow shows how the screws are positioned in an angulated configuration, forming the “tent-like” structure characteristic of the tent-pole technique. (E) Biomaterial placement. (F) Membrane fixation with tacks; the arrow shows how the chosen fixation method keeps the membrane firmly stable in position. (G) Verification of graft stability. (H) Immediate postoperative view; the arrow indicates the horizontal mattress sutures. (I) Postoperative view at 12 days. (J) Postoperative view at 80 days.

Pharmacological treatment included amoxicillin with clavulanic acid 875/125 mg every 12 hours for seven days, ibuprofen 600 mg every eight hours for five days, injectable dexamethasone 8 mg/2 mL in a single dose on the day of the procedure, and 0.12% chlorhexidine mouthwash in 20 mL undiluted rinses for one minute every 12 hours. One month later, a transitional prosthesis was placed to provide occlusal, phonetic, and esthetic stability (Figure 5).

**Figure 5:**
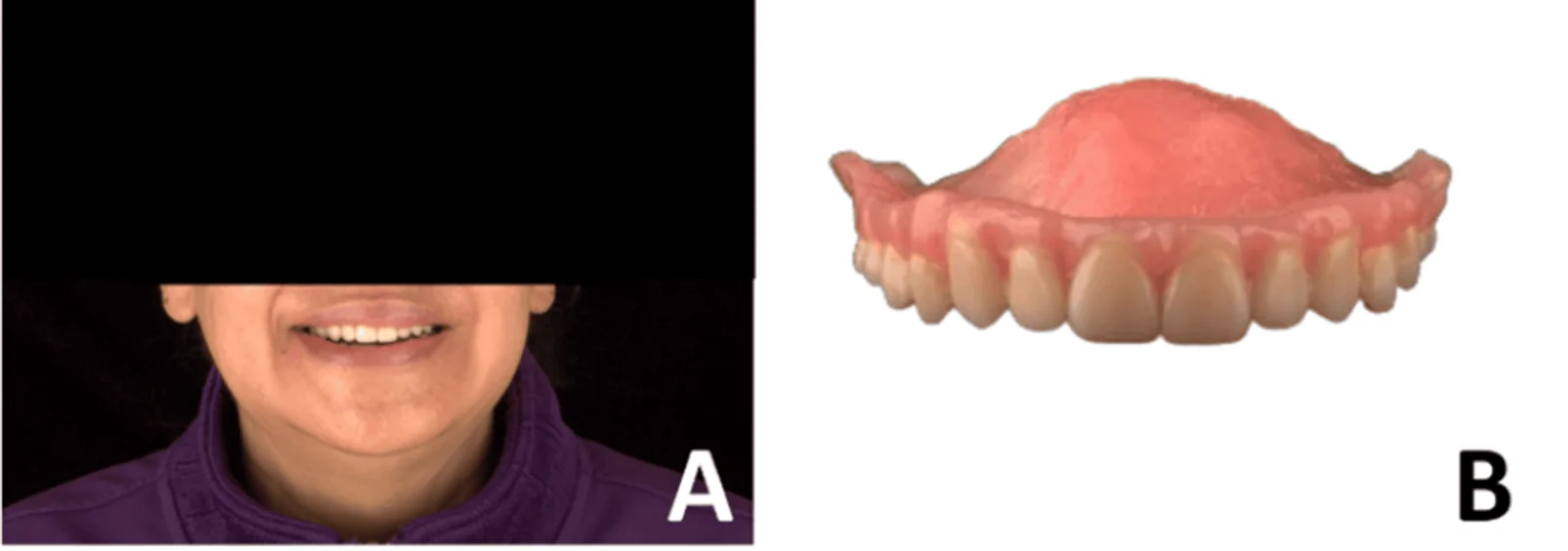
Transitional prosthesis. (A) Frontal facial view of the patient wearing the transitional prosthesis; (B) frontal intraoral view of the transitional prosthesis in position.

The Angle Class III intermaxillary relationship and the need to restore upper lip support confirmed the indication of the previously described bar-supported overdenture, with two splinted bars, one on each side of the arch. For implant planning, five light-polymerized acrylic resin markers (Palatray, Kulzer, Hanau, Germany) were placed on an acrylic duplicate of the prosthesis with a previously verified fit. Two CBCT scans were then obtained under standardized conditions: one of the patient wearing the marked prosthesis and a second one of the marked prosthesis alone. The two datasets were subsequently superimposed using planning software and the shared radiopaque references, which allowed the prosthetic design to be virtually integrated with the underlying bone anatomy and each implant to be planned in a prosthetically driven manner (Figure 6).

**Figure 6:**
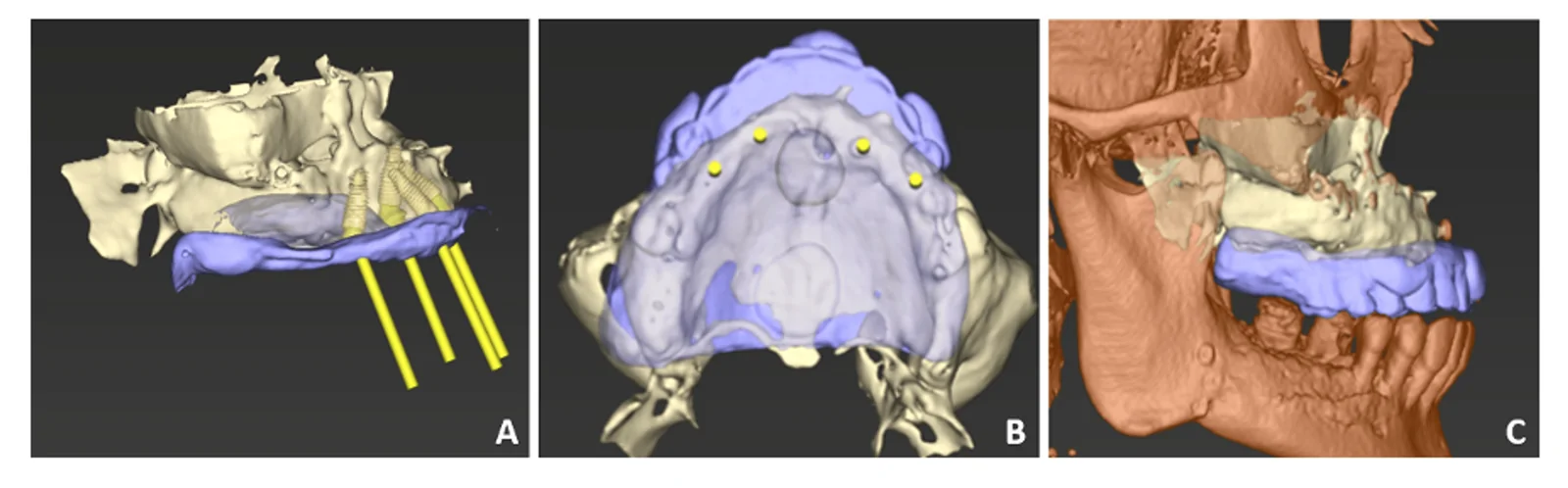
Dual-scan cone beam computed tomography (CBCT) implant planning. (A) Implant planning using a dual-scan technique. (B, C) Registration of DICOM files for prosthetically guided implant planning. DICOM: Digital Imaging and Communications in Medicine

The DICOM files were imported into the planning software (coDiagnostiX; Dental Wings, Chemnitz, Germany). The implants were planned at sites #14 (4.0 × 10 mm), #12 (3.5 × 10 mm), #22 (4.0 × 8 mm), and #24 (4.0 × 10 mm) (GM Helix Acqua; Neodent, Curitiba, Brazil), and a bone-supported stereolithographic surgical guide with fixation pins was fabricated. This guide was fully bone-supported, rested directly on the exposed alveolar bone after full-thickness flap elevation, and was stabilized with bone pins prior to the osteotomy sequence. The implants were placed with a final torque of 30 Ncm. Localized peri-implant GBR was then performed at sites #12 and #14 to address minor buccal peri-implant defects observed at the time of implant placement, using an allograft (OraGRAFT 70/30 Cortical) covered with a resorbable collagen membrane (OSSIX Plus 30 × 40 mm; Datum Dental, Lod, Israel) sutured to the periosteum. It should be noted that this localized peri-implant regeneration is different from the previous horizontal ridge augmentation of the anterior maxilla and was strictly limited to the peri-implant fenestrations observed at the moment of implant placement. The ridge was then covered with plasma rich in growth factors (PRGF; BTI Biotechnology Institute, Vitoria, Spain) and sutured with 4-0 nylon following the same protocol. Finally, the implant position was radiographically verified (Figure 7).

**Figure 7:**
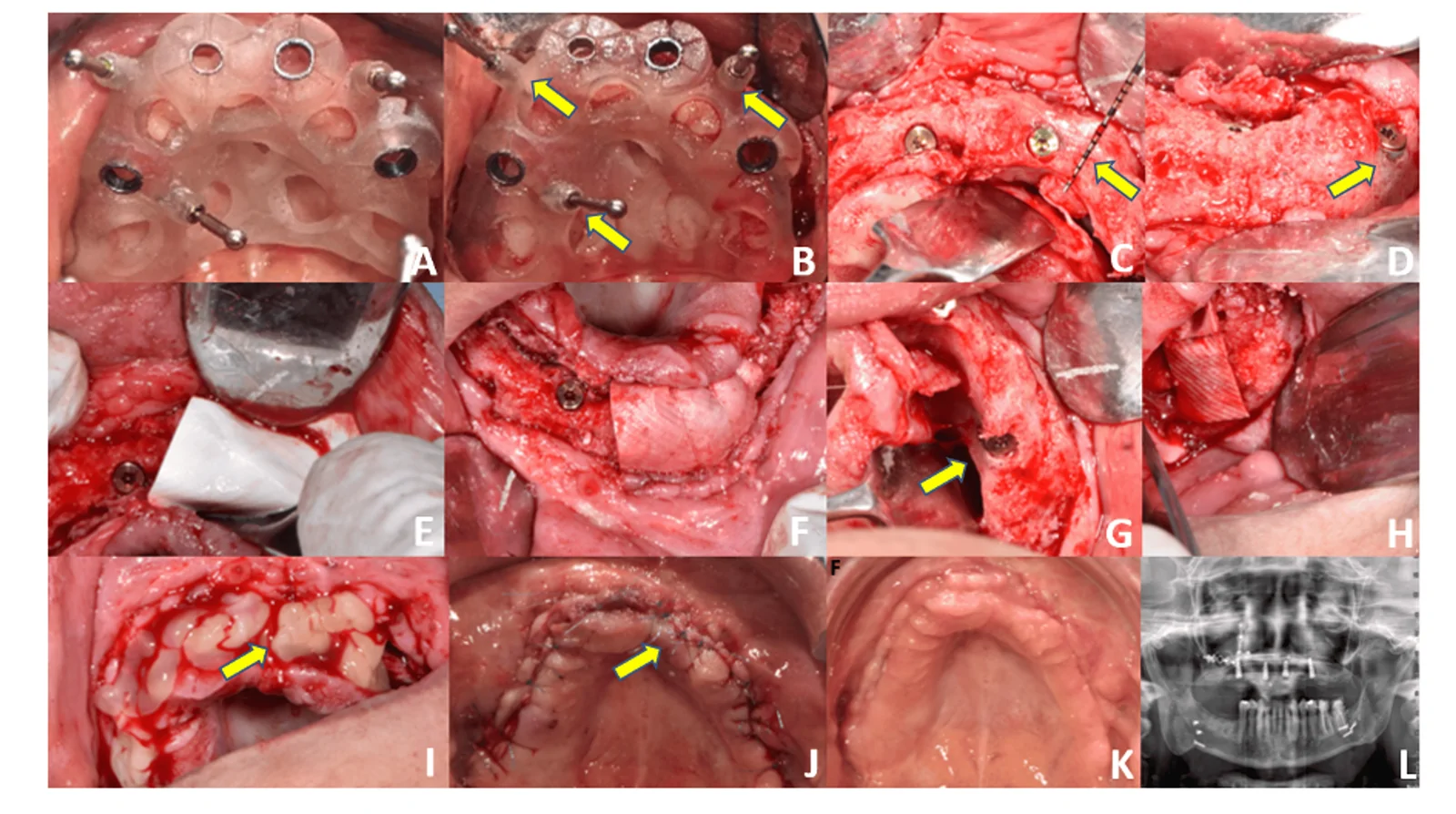
Simultaneous implant placement and localized peri-implant guided bone regeneration (GBR). (A) Verification of guide placement. (B) Full-thickness flap, placement, and stabilization of the guide; the arrow indicates the inserted bone pins. (C) Implant placement; the arrow shows the probe indicating the previously regenerated Seibert Type III defect. (D) Buccal peri-implant defect after placement of implant #12; the arrow indicates the defect to be regenerated. (E) GBR in the buccal area of implant #12. (F) Membrane sutured to the periosteum and verification of graft stability. (G) Buccal peri-implant defect after placement of implant #14; the arrow indicates the defect to be regenerated. (H) GBR in the palatal area of implant #14, membrane sutured to the periosteum, and verification of graft stability. (I) Harvesting and placement of PRGF clots; the arrow indicates the clots in the surgical bed. (J) Immediate postoperative view; the arrow indicates the horizontal mattress sutures. (K) Healing at 30 days. (L) Radiographic verification 20 days after the procedure.

In the lower arch, the implant at #46 was explanted, and tooth #47 was extracted. The ridge was preserved with a xenograft (InterOss 0.25-1 mm; SigmaGraft) covered with a resorbable membrane (OSSIX Plus 30 x 40 mm; Datum Dental) sutured to the periosteum with polyglactin 910 4-0 (Vicryl), and the site was closed with the same suture pattern (Figure 8).

**Figure 8:**
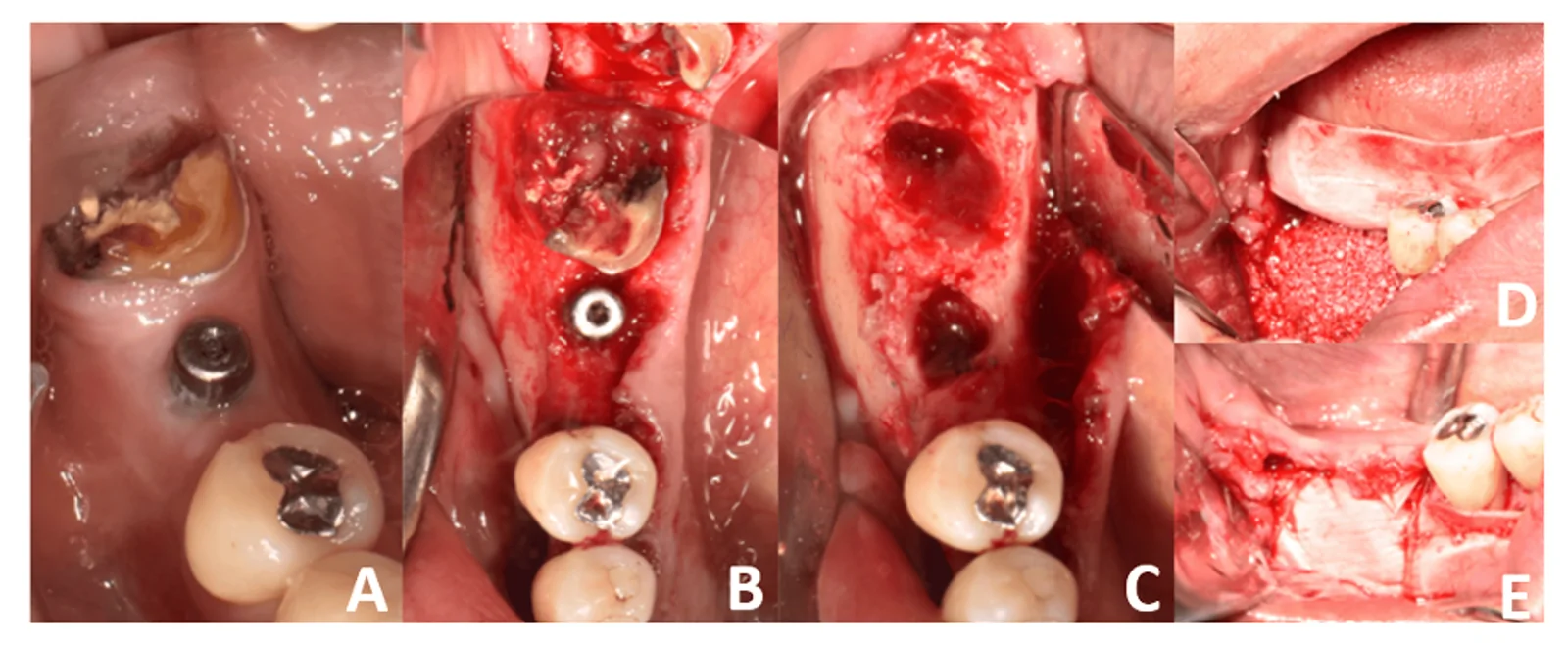
Mandibular ridge preservation. (A) Initial condition. (B) Explantation of the implant at site #46 and extraction of tooth #47. (C) Residual horizontal defect. (D) Biomaterial placement. (E) Membrane sutured to the periosteum.

Six months later, a single implant was planned and placed at site #46 (GM Helix Acqua, 4.3 × 10 mm; Neodent, Curitiba, Brazil), with a simultaneous graft of de-epithelialized connective tissue harvested from the palate to improve the peri-implant keratinized mucosa. Four months later, the implant was restored with a screw-retained zirconia crown (Figure 9).

**Figure 9:**
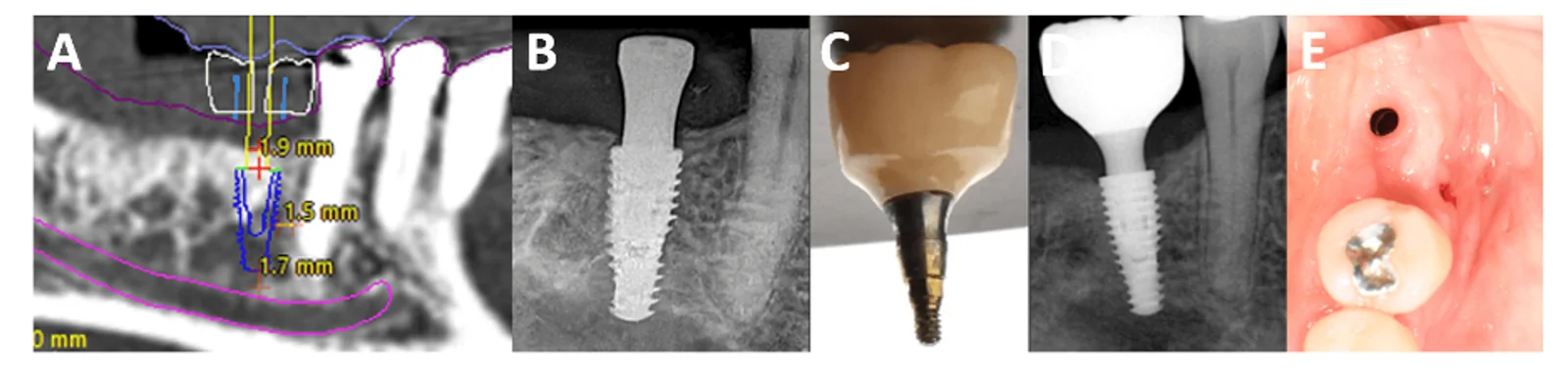
Rehabilitation of site #46. (A) Implant planning. (B) Implant placement and radiographic verification. (C) Screw-retained zirconia crown. (D) Radiographic verification of the crown. (E) Stable peri-implant soft tissues.

In the posterior sector of the third quadrant, crown lengthening with simultaneous core build-up and zirconia crowns was performed (Figure 10).

**Figure 10:**
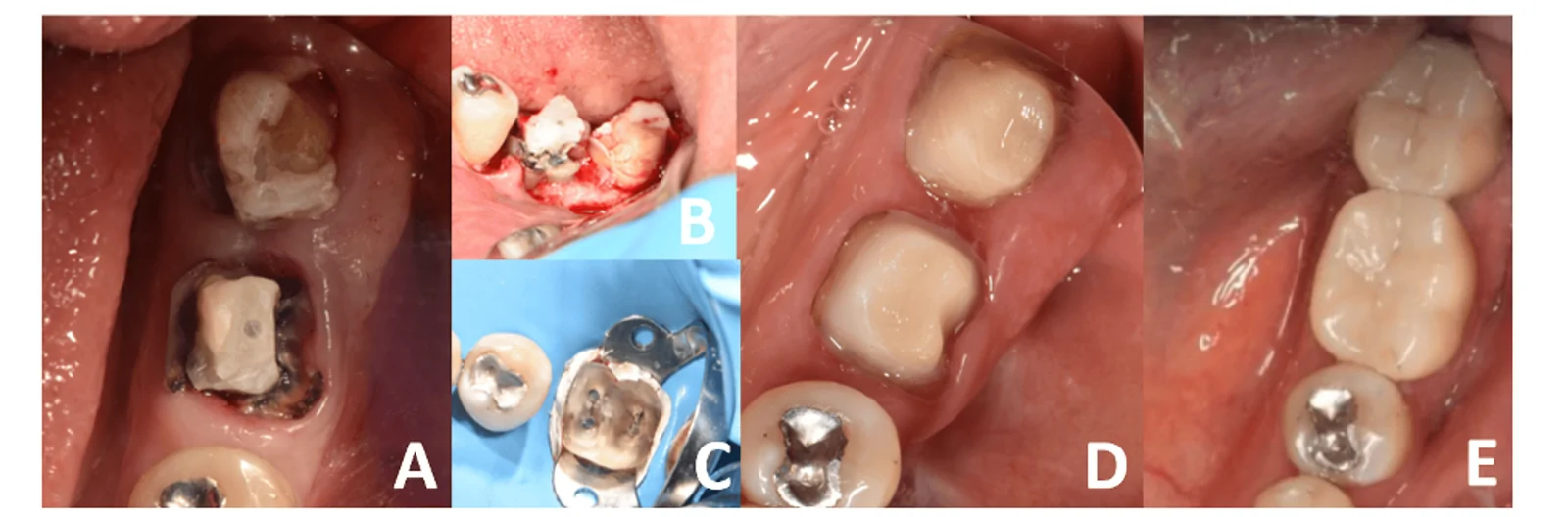
Rehabilitation of the left posterior mandible. (A) Initial condition. (B) Crown lengthening surgery. (C) Immediate core build-up with a core resin (ParaCore; Coltène/Whaledent, Altstätten, Switzerland). (D) Crown preparations. (E) Cementation of zirconia crowns.

After the lower arch was completed, an analog implant-level impression was taken with polyvinyl siloxane, and its accuracy was verified using a stone jig. The maxillary wax occlusion rim and intermaxillary records were obtained, splinted resin bars were fabricated for each side, and their passive fit was checked. After the tooth try-in, the definitive titanium bars and the definitive acrylic overdenture were fabricated and delivered on the definitive cast (Figure 11).

**Figure 11:**
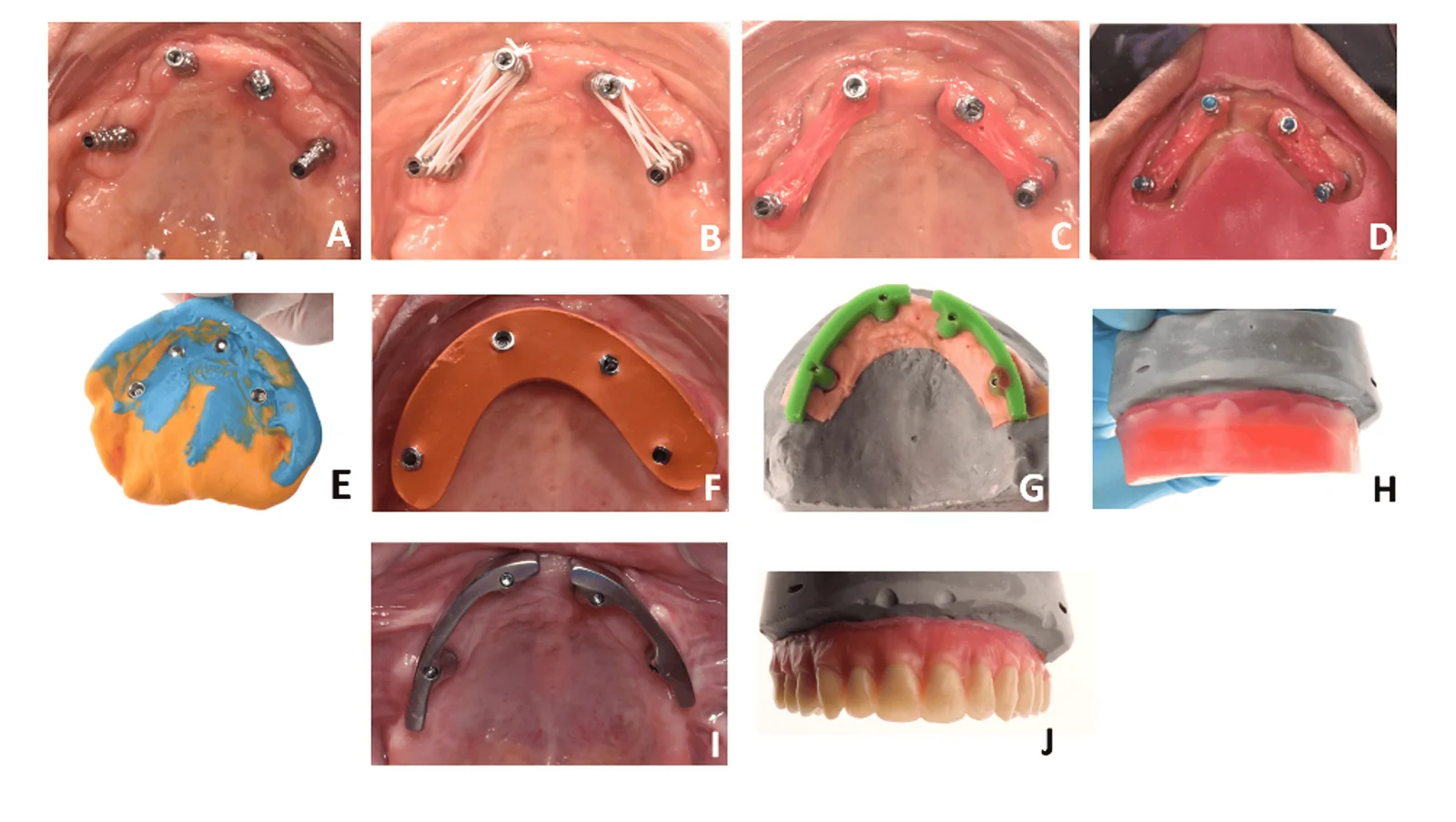
Prosthetic workflow. (A) Placement of impression copings. (B) Initial splinting with dental floss. (C, D) Application of low-shrinkage pattern resin (Pattern Gel; Ideas Dentales, Mexico City, Mexico), sectioned to compensate for shrinkage, reconnected, and used to verify the custom tray (Palatray; Kulzer). (E) Final polyvinyl siloxane impression (Elite; Zhermack, Badia Polesine, Italy). (F) Verification jig in Type IV stone. (G) Fabrication and try-in of the resin bar patterns. (H) Occlusal-rim try-in. (I) Fabrication and try-in of the titanium bars. (J) Completed implant-retained overdenture.

After delivery of the definitive prosthesis, the VDO was re-established at a clinically satisfactory level, and the residual Class III intermaxillary relationship was preserved and compensated through the design of the maxillary overdenture, including its buccal flange and its occlusal scheme. Neither the bone augmentation phases nor the implant placement modified the intermaxillary relationship; this relationship was addressed exclusively at the prosthetic level, as originally planned.

CBCT-based assessment of dimensional changes

To complement the surgical description, a quantitative assessment of alveolar ridge dimensions was performed on the pre-treatment CBCT and on a control CBCT obtained nine months after the first regenerative intervention. Both datasets were superimposed in Exocad software (exocad GmbH, Darmstadt, Germany). The highest point of the ridge was used as the reference to measure the buccopalatal/lingual width at 1, 3, and 5 mm in the apical direction (Figure 12). For height measurements, the highest points of the buccal and palatal ridges were used as references; a perpendicular line was drawn, and the buccal, middle, and palatal heights were measured until they intersected with the perpendicular line representing the apex. In the edentulous areas, the apex of the adjacent tooth was used as a reference, whereas in the control CBCT, the length of the previously existing alveolar socket in the same region was used (Figure 12). Regional means were then obtained by averaging, within each anatomical region (anterior maxillary tent-pole area, posterior maxillary ridge-preservation area, and mandibular ridge-preservation area), the site-specific measurements collected at every treated position. Because the study involves a single patient, these means are presented as descriptive within-case values, and no measure of dispersion is reported.

**Figure 12:**
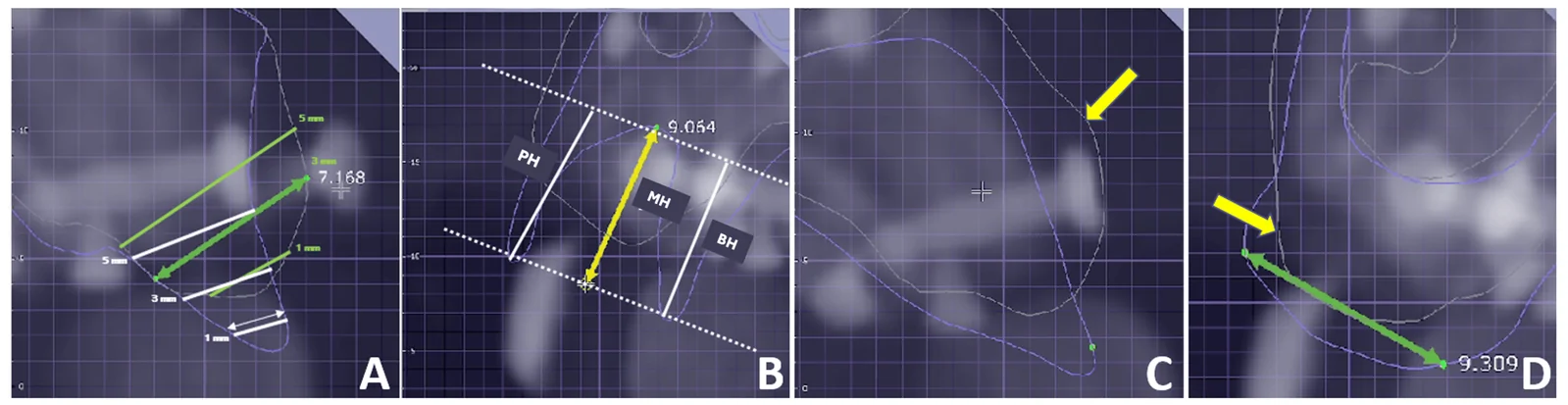
CBCT measurement protocol. (A) Buccopalatal/lingual width measured at 1, 3, and 5 mm apical to the highest point of the osseous crest. (B) Bone height measured from the crest to the apical reference at three positions: buccal height (BH), middle height (MH), and palatal height (PH). (C) Superimposition of baseline and postoperative scans at the anterior maxillary tent-pole site, showing buccopalatal gain; the arrow indicates the ridge increase. (D) Superimposition of baseline and postoperative scans at the posterior maxillary ridge-preservation site, showing apicocoronal resorption with preserved width; the arrow indicates bone-height loss. CBCT: cone-beam computed tomography

The regional dimensional changes observed in this patient are summarized in Table 1. It should be acknowledged that, in a single-patient case report, these values are intended to illustrate the regional pattern of dimensional change observed in the presented case, obtained by within-patient superimposition of the baseline and nine-month CBCT scans, and are not intended to provide population-level estimates. Therefore, no measure of dispersion is reported, and the results should not be extrapolated to other clinical scenarios.

**Table 1:** Mean regional changes in the ridge dimensions (mm). Mean values in millimeters, obtained by superimposing the baseline and nine-month scans in the same patient (descriptive within-case measurements). Width was measured at 1, 3, and 5 mm apical to the crest, and height was measured at the buccal (BH), palatal or lingual (PH/LH), and middle (MH) positions. A dash (-) indicates no change in the direction or location. GBR: guided bone regeneration

Region	Change	1 mm	3 mm	5 mm	BH	PH/LH	MH
Maxillary ridge preservation	Resorption	1.55	1.24	0.70	2.07	2.17	1.63
	Gain	-	0.48	0.86	-	-	1.54
Tent-pole GBR	Gain	2.78	3.27	3.36	-	-	-
	Resorption	4.85	1.10	0.04	2.61	3.05	2.35
Mandibular ridge preservation	Resorption	0.59	0.39	0.56	-	-	-
	Gain	-	-	-	0.76	2.34	1.48

In this patient, maxillary ridge preservation showed a gradient: resorption predominated near the crest (mean of 1.55 mm at 1 mm and 1.24 mm at 3 mm) and decreased apically, up to a mean gain of 0.86 mm at 5 mm; in height, resorption predominated as well (2.07 mm buccal, 2.17 mm palatal, and 1.63 mm mid-ridge). In the mandible (sites #46 and #47), the same point measurements indicated width resorption of 0.59, 0.39, and 0.56 mm at 1, 3, and 5 mm, respectively, whereas only a height gain was recorded (0.76 mm buccal, 2.34 mm lingual, and 1.48 mm mid-ridge). In the tent-pole GBR area (sites #11, #21, #22, and #23), width gains were observed at #11, #21, and #22 (mean of 2.78, 3.27, and 3.36 mm at 1, 3, and 5 mm, respectively), with localized resorption at #23 (4.85, 1.10, and 0.04 mm at 1, 3, and 5 mm, respectively); in terms of height, only resorption was recorded (2.61 mm buccal, 3.05 mm palatal, and 2.35 mm mid-ridge).

Overall, this regional assessment confirmed that the horizontal component of the atrophy in the anterior maxilla was the main anatomical limitation for implant-supported rehabilitation and directly justified the tent-pole GBR approach adopted in this case, as well as the prosthetically driven implant planning based on the dual-scan CBCT protocol (Figure 13).

**Figure 13:**
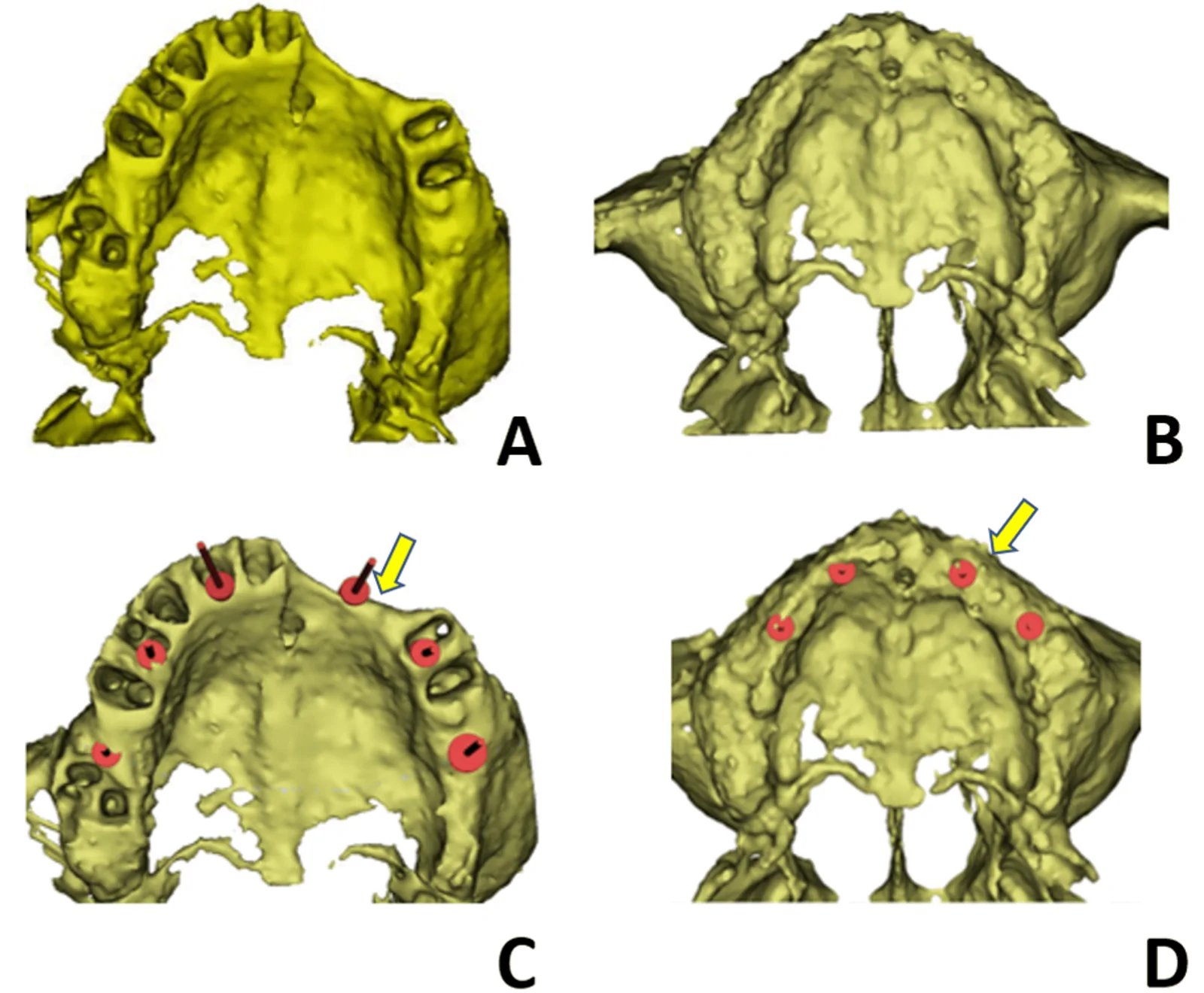
Effect of regeneration on implant planning, illustrated on cone-beam computed tomography (CBCT) three-dimensional volumetric reconstructions. (A) Initial condition. (B) Tomographic comparison of ridge dimensions at nine months. (C) Initial implant planning, where inadequate bone volume precluded placement; the arrow indicates a Seibert Type III defect. (D) Planning at nine months, showing sufficient bone volume for three-dimensionally stable implant placement; the arrow indicates the regenerated region.

## Discussion

In ridge preservation, both areas of resorption and gain occur, which is consistent with the biological variability of the alveolar healing process. In contrast, when the graft was stabilized with tenting screws following the tent-pole technique, the increase in width was more marked, whereas the changes obtained in the vertical dimension were less evident.

Several studies have supported the use of the tent-pole technique for ridge augmentation. Guillen et al. [8] conducted a prospective, randomized, split-mouth study on anterior maxillary defects in 10 patients with missing upper incisors and ridge widths of 2-3.5 mm and compared conventional and wide-head screws. They used xenografts (Lumina Porous 1000-2000 µm; Criteria Biomateriais, São Paulo, Brazil) hydrated with PRGF and covered with a fibrin membrane. The mean width increase with standard screws was 1.05 mm at the crest and 2.45 and 2.70 mm at 5 and 10 mm, respectively; with wide-head screws, it was 1.50 mm at the crest and 3.10 and 3.45 mm at 5 and 10 mm, respectively. Although wide-head screws performed slightly better, the differences were not statistically significant.

In our case, the values obtained in the areas treated with tenting screws, xenograft (InterOss), and autograft covered with a porcine collagen membrane (Intercollagen Guide 30 × 40 mm; SigmaGraft) were broadly comparable to those described by that group at #11, #21, and #22 (mean of 2.78 mm at 1 mm, 3.27 mm at 3 mm, and 3.36 mm at 5 mm, respectively), with localized resorption at #23 (4.85, 1.10, and 0.04 mm at 1, 3, and 5 mm, respectively). This comparison should be interpreted as an illustrative descriptive parallel between a single-case observation and a controlled clinical study, rather than as evidence of equivalence.

Chacón et al. [9] reported horizontal and vertical gains in anterior maxillary defects (eight patients) and anterior mandibular defects (two patients). They standardized autologous bone with a xenograft (50:50) (Bio-Oss; Geistlich Pharma, Wolhusen, Switzerland) and a membrane (OSSIX Volumax; Datum Dental, Lod, Israel) using one to four screws, depending on the defect. All sites formed bone, with a correction of 5-9 mm and a mean vertical gain of 6.2 ± 1.61 mm at 12 months. In the only case with flap exposure, the gain was 6 mm over a 12 mm defect. These reports suggest that the arrangement of the screws within the defect may be more relevant than their type: they should be oriented toward the area to be regenerated, and their position depends on the morphology of the defect.

Deeb et al. [10] compared the tent-pole method with other horizontal augmentation techniques and reported comparable or superior outcomes in terms of graft regeneration and stability, and Durrani et al. [11] described favorable space-maintenance and volumetric behavior provided by these screws during GBR.

The use of biomaterials in ridge preservation has considerable support. In a systematic review by Tan et al. [12], naturally healed alveoli showed considerable resorption, particularly buccal (approximately 4 mm in width and greater than 2 mm in height). With preservation, the loss was reduced by half, retaining an additional 1.5 to 2 mm in width and nearly 1 mm more in height. No biomaterial was superior; however, all methods improved ridge stability compared to spontaneous healing. In our case, despite some resorption, there were also areas of gain, and the loss was less than expected without intervention, a pattern consistent with the findings of Avila-Ortiz et al. [13] and Corbella et al. [14].

In the maxilla, allografts and xenografts were combined to draw on the properties of both. Our results revealed a transition from crestal resorption (mean of 1.55 mm at 1 mm) to basal gain (0.86 mm at 5 mm apical). In terms of height, resorption predominated in both the buccal (2.07 mm) and palatal (2.17 mm) regions, and in the middle region, resorption (1.63 mm) had values similar to those of gain (1.54 mm).

The use of allografts as the main component supplemented with adjacent autografts is supported by their osteoconductive and osteoinductive properties and the reduction in morbidity associated with a second donor site. Nelson and Mealey [15] studied the effect of healing time after preservation with a 70% mineralized allograft (FDBA) and 30% demineralized allograft (DFDBA) and compared healing periods of 8-10 and 18-20 weeks. Ridge loss was approximately 1 mm in width and height, with no differences between time periods. Sites with longer healing times contained nearly twice as much vital bone and less residual material, indicating more advanced maturation.

In a randomized trial, Poulias et al. [16] compared preservation with allograft alone versus allograft with a layer of xenograft, both under a polylactide membrane. The allograft alone lost 1.6 mm of crestal width, in contrast to 0.3 mm with the xenograft overlay; at 5 mm apical, the control lost 0.7 mm, and the overlay group gained 0.5 mm. The addition of a xenograft better preserved the ridge width.

In the mandible, only a xenograft was used, with favorable results that allowed the placement of an implant with satisfactory osseointegration and survival of the implant-prosthesis unit at one year of follow-up. A review by Sah et al. [17] revealed that, compared with spontaneous healing, xenograft alone preserves between 1.5 and 2.5 mm of additional width and between 2.5 and 3 mm of buccal height, with 10% more vital bone than spontaneous healing.

Regarding implants in regenerated sites, Hong et al. [18] compared 264 implants in native bone and 133 in regenerated bone, with similar cumulative survival rates (97.5% versus 96.4%), and smoking was the main failure factor. Hämmerle et al. [19] reported a 90% survival rate at regenerated sites after at least one year of function, and Clementini et al. [20] reported success rates between 61.5% and 100%, above 90% in most related studies. Unlike these series, the present case report describes a single implant in regenerated bone with a short follow-up period; these rates, therefore, provide context but cannot be extrapolated to the present case.

From a clinical standpoint, the sequence adopted in this case suggests that, in patients with severe anterior maxillary defects and previously failed regenerative attempts, a staged approach combining alveolar ridge preservation, tent-pole GBR, and prosthetically driven implant placement based on a dual-scan CBCT protocol can restore bone volume sufficient for implant-supported rehabilitation and enable the delivery of an implant-retained overdenture that additionally addresses lip support, biomechanical control, and hygiene in the context of a Class III intermaxillary relationship. These observations, however, are preliminary and should be interpreted within the scope of a single-case description.

This case report has several limitations that are inherent to its design and that should be considered when interpreting the reported outcomes. First, it presents a single patient, so all dimensional changes described here are within-patient descriptive observations and cannot be extrapolated to a broader population; consequently, no statistical significance testing was performed, and the values reported in Table 1 should be understood as descriptive within-case measurements rather than inferential estimates. Second, the volumetric assessment was performed by superimposing two CBCT scans acquired at different time points, a procedure that is subject to operator- and registration-dependent variability. Although a standardized methodology and consistent anatomical landmarks were used, no formal intra- or inter-operator reliability testing was applied to the present dataset, and this constitutes a methodological limitation that future studies should address through repeated measurements and calibrated observers. Third, baseline and nine-month CBCT scans define the timeframe for the dimensional assessment, whereas the follow-up period after final prosthetic loading is still short. Comprehensive long-term functional and esthetic outcomes, including standardized intraoral photographic documentation of the definitive rehabilitation in function, are being collected in an ongoing prospective follow-up and will be reported in a subsequent publication. Finally, larger case series and prospective studies with longer follow-up periods and formal reliability testing of the CBCT superimposition method are required to confirm the trends observed in the present case.

## Conclusions

In the present case, the regenerative techniques employed were associated with clinically favorable outcomes. Ridge preservation was accompanied by less contraction than would be expected without intervention, which is consistent with the role attributed to the technique in the literature in attenuating post-extraction remodeling. The tent-pole technique allowed the treatment of a Seibert Class III defect, with a within-case width gain of up to 3.36 mm that was sufficient in this patient for three-dimensional implant positioning and for the delivery of the planned prosthetic rehabilitation. Finally, prosthetically guided planning (reverse planning) articulated the surgical and prosthetic phases in a coordinated workflow; long-term dimensional stability and functional performance are being documented in an ongoing follow-up.

Similarly, the selection of biomaterials according to the characteristics of each defect, along with the stable fixation of the graft and the barrier membrane, appears to have contributed to the stability of the regenerated volume. These observations are specific to a single patient, were obtained through within-case descriptive measurements without formal statistical testing, and cover a limited follow-up period after prosthetic loading; therefore, they should be interpreted as illustrative of the clinical sequence adopted rather than as evidence of generalizable predictability and require confirmation in larger case series and prospective studies with longer follow-up periods.
